# What the European Reference Network Registry for Rare Liver Diseases Tells Us About Primary Biliary Cholangitis in European Practice

**DOI:** 10.1002/ueg2.70143

**Published:** 2025-11-17

**Authors:** Marten A. Lantinga

**Affiliations:** ^1^ Department of Gastroenterology and Hepatology Amsterdam UMC University of Amsterdam Amsterdam the Netherlands

Primary biliary cholangitis (PBC) continues to evolve as a disease, due to advances in non‐invasive options to monitor disease progression, new therapeutic strategies, and identification of predictors for biochemical response. [1] As illustrated by a recent nationwide Dutch cohort study, the epidemiology of PBC is evolving reflected by a rising incidence, with the highest point prevalence increase observed amongst those aged 65 years and older. [2] The study by Gerussi et al. in this issue of the United European Gastroenterology Journal provides a prospective snapshot of the care provided for 327 patients across six European Reference Network Registry for Rare Liver Diseases (ERN R‐LIVER) referral centers. [3] The prospective multicenter design of this study represents a methodological strength that distinguishes it from previous PBC patient registries. By including patients diagnosed between 2017 and March 2024, the authors can reflect on the current approach and challenges faced in these patients. This offers insights into how practice has followed guidelines, while on the other hand illustrating the gap in treatment options for patients not responding to either ursodeoxycholic acid (UDCA), off‐label fibrates, or other strategies including for example obeticholic acid (OCA).

A noteworthy finding is that almost 90% of PBC patients in the studied cohort had no signs of cirrhosis on imaging at diagnosis, further underlining the shift from historical studies where advanced disease at presentation was common. The widespread implementation of performing liver stiffness measurement (LSM) in 70.3% of patients at diagnosis is a relevant observation, as a recent international multicenter study analyzing 1.793 PBC patients (followed over a median of 22 months) showed that discordance between LSM and biochemical response is frequent, and that the current LSM value is the strongest predictor of future liver‐related events in these patients. [4] Moreover, as shown in a multicenter Portuguese cohort study, among others baseline presence of cirrhosis was associated with a risk of incomplete response to UDCA, therefore warranting close monitoring of these patients when identified with cirrhosis at baseline. [5].

The almost universal initiation of first‐line UDCA treatment at diagnosis (92.4%) at the recommended dose (median 13.3 mg/kg per day) shows the current near‐perfect adherence to the clinical practice guideline across the included European centers. [6] Despite that inadequate biochemical response was observed in only 14.9% (Toronto criteria) and 22.3% (POISE criteria) of patients, respectively, normalization of alkaline phosphatase (ALP), was seen in 49.5% of patients after 12 months of UDCA. In contrast to this, the authors state that less than half of those with an inadequate response (following the Toronto criteria) were treated with any second‐line treatment. Two main reasons could account for this. First, differences in availability and/or reimbursement of OCA across European countries exist, and fibrates remain an off‐label option only. Second, as discussed in detail by the authors themselves, a combination of excluding patients participating in a clinical study investigating new treatment options (e.g., elafibranor and seladelpar), unavailability/delayed practice implementation of evidence at the time (e.g., the results of the BEZURSO trial [7]), and lack of specific treatment considerations in the current practice guideline, could have resulted in the relatively low number of patients receiving any form of second‐line therapy.

On the other hand, despite the prospective nature of the study by Gerussi et al. incomplete follow‐up data led to the exclusion of 56% (*n* = 439/790) of patients of the total PBC cohort, potentially biasing these numbers. This exclusion rate is however remarkably similar to a recent retrospective study performed in The United States, in which only 52% of patients received adequate biochemical monitoring of treatment response. [8] This could highlight the unmet need for optimizing the biochemical monitoring and follow‐up of treatment response of PBC patients. The importance of adequate follow‐up is further highlighted by a recent Canadian multicenter cohort study which showed that loss of biochemical response at any time worsens outcomes in UDCA‐treated patients with PBC. [9].

While the authors primarily focus on biochemical response, the study of Gerussi et al. fails to address the substantial symptom burden patients can experience. For example, fatigue is being increasingly recognized as a major determinant of health‐related quality of life in PBC patients, reflected by the position paper by the ERN R‐LIVER group which proposes an algorithm for its diagnosis and management. [10] Future registries should therefore integrate patient‐reported outcome measures with biochemical monitoring to ensure a meaningful improvement in quality of life.

To conclude, while PBC management has advanced with improved early detection and guideline adherence for first‐line UDCA treatment, significant gaps persist in monitoring biochemical response and addressing symptom burden.

## Funding

The author has nothing to report.

## Conflicts of Interest

The author declares no conflicts of interest.

## Data Availability

The author has nothing to report.

## References

[1] A. Laschtowitz , R. C. de Veer , A. J. Van der Meer , and C. Schramm , “Diagnosis and Treatment of Primary Biliary Cholangitis,” United European Gastroenterol J 8, no. 6 (2020): 667–674, 10.1177/2050640620919585. PMC7437077 32299307

[2] R. C. de Veer , M. C. B. van Hooff , E. Werner , et al., “Incidence and Prevalence of Primary Biliary Cholangitis in the Netherlands ‐ a Nationwide Cohort Study,” JHEP Rep 6, no. 8 (2024): 101132, 10.1016/j.jhepr.2024.101132. 39113899 PMC11304051

[3] A. Gerussi , E. Nofit , D. P. Bernasconi , et al., “Trends in Primary Biliary Cholangitis: Prospective Cohort Study From the European Reference Network Registry (R‐LIVER),” United European Gastroenterol J (2025): 70134, 10.1002/ueg2.70134. PMC12704578 41124063

[4] Y. J. Wong , L. Lam , P. A. Soret , et al., “Prognostic Value of Liver Stiffness Measurement Vs. Biochemical Response in Primary Biliary Cholangitis,” Journal of Hepatology (2025), 10.1016/j.jhep.2025.09.024. 41047080

[5] H. Cortez‐Pinto , R. Liberal , S. Lopes , et al., “Predictors for Incomplete Response to Ursodeoxycholic Acid in Primary Biliary Cholangitis. Data From a National Registry of Liver Disease,” United European Gastroenterol J 9, no. 6 (2021): 699–706, 10.1002/ueg2.12095. PMC8280809 34102008

[6] EASL Clinical Practice Guidelines . “The Diagnosis and Management of Patients With Primary Biliary Cholangitis,” Journal of Hepatology 67, no. 1 (2017): 145–172, 10.1016/j.jhep.2017.03.022. 28427765

[7] C. Corpechot , O. Chazouillères , A. Rousseau , et al., “A Placebo‐Controlled Trial of Bezafibrate in Primary Biliary Cholangitis,” New England Journal of Medicine 378, no. 23 (2018): 2171–2181, 10.1056/nejmoa1714519. 29874528

[8] S. C. Gordon , A. Sahota , M. A. Schmidt , et al., “Assessment of Adherence to Guidelines for Biochemical Monitoring and Ursodeoxycholic Acid Treatment Response in a Retrospective Cohort of US Patients With Primary Biliary Cholangitis,” BMJ Open Gastroenterol 12, no. 1 (2025): e001899, 10.1136/bmjgast-2025-001899. PMC12548585 41125399

[9] S. B. Roberts , W. J. Choi , L. Worobetz , et al., “Loss of Biochemical Response at Any Time Worsens Outcomes in UDCA‐Treated Patients With Primary Biliary Cholangitis,” JHEP Rep 6, no. 10 (2024): 101168, 10.1016/j.jhepr.2024.101168. 39380718 PMC11460452

[10] O. M. Koc , A. K. Toussaint , A. Untas , et al., “Fatigue in People With Primary Biliary Cholangitis: A Position Paper From the European Reference Network for Rare Liver Diseases,” Lancet Gastroenterology & Hepatology (2025), 10.1016/S2468-1253(25)00257-2. 41082897

